# Efficacy of adipose tissue-derived stem cells in locomotion recovery after spinal cord injury: a systematic review and meta-analysis on animal studies

**DOI:** 10.1186/s13643-021-01771-w

**Published:** 2021-07-31

**Authors:** Seyedeh Niloufar Rafiei Alavi, Arian Madani Neishaboori, Hasti Hossein, Arash Sarveazad, Mahmoud Yousefifard

**Affiliations:** 1 Physiology Research Center, Iran University of Medical Sciences, Hemmat Highway, P.O Box: 14665-354, Tehran, Iran; 2 Colorectal Research Center, Iran University of Medical Sciences, Niayesh St, Satarkhan Av, P.O Box: 14665-354, 1449614535 Tehran, Iran; 3 Nursing Care Research Center, Iran University of Medical Sciences, Tehran, Iran

**Keywords:** Stem cell, Spinal cord injuries, Animal study, Meta-analysis

## Abstract

**Background:**

Considerable disparities exist on the use of adipose tissue-derived stem cells (ADSCs) for treatment of spinal cord injury (SCI). Hence, the current systematic review aimed to investigate the efficacy of ADSCs in locomotion recovery following SCI in animal models.

**Methods:**

A search was conducted in electronic databases of MEDLINE, Embase, Scopus, and Web of Science until the end of July 2019. Reference and citation tracking and searching Google and Google Scholar search engines were performed to achieve more studies. Animal studies conducted on rats having SCI which were treated with ADSCs were included in the study. Exclusion criteria were lacking a non-treated control group, not evaluating locomotion, non-rat studies, not reporting the number of transplanted cells, not reporting isolation and preparation methods of stem cells, review articles, combination therapy, use of genetically modified ADSCs, use of induced pluripotent ADSCs, and human trials. Risk of bias was assessed using Hasannejad et al.’s proposed method for quality control of SCI-animal studies. Data were analyzed in STATA 14.0 software, and based on a random effect model, pooled standardized mean difference with a 95% confidence interval was presented.

**Results:**

Of 588 non-duplicated papers, data from 18 articles were included. Overall risk of bias was high risk in 8 studies, some concern in 9 studies and low risk in 1 study. Current evidence demonstrated that ADSCs transplantation could improve locomotion following SCI (standardized mean difference = 1.71; 95%CI 1.29–2.13; *p* < 0.0001). A considerable heterogeneity was observed between the studies (*I*^2^ = 72.0%; *p* < 0.0001). Subgroup analysis and meta-regression revealed that most of the factors like injury model, the severity of SCI, treatment phase, injury location, and number of transplanted cells did not have a significant effect on the efficacy of ADSCs in improving locomotion following SCI (*p*_for odds ratios_ > 0.05).

**Conclusion:**

We conclude that any number of ADSCs by any prescription routes can improve locomotion recovery in an SCI animal model, at any phase of SCI, with any severity. Given the remarkable bias about blinding, clinical translation of the present results is tough, because in addition to the complexity of the nervous system and the involvement of far more complex motor circuits in the human, blinding compliance and motor outcome assessment tests in animal studies and clinical trials are significantly different.

**Supplementary Information:**

The online version contains supplementary material available at 10.1186/s13643-021-01771-w.

## Background

Following spinal cord injury (SCI), a cascade of reactions occurs in the injured area, all of which can damage the nerve tissue. This nerve damage disrupts the neural connection between higher and lower parts of the injury; thus, it seems that the disabilities symptoms will persist until the affected area is healed and the active synapses between the upper and lower part of the injured spinal cord are restored [1, 2].

Today, cell transplantation is thought to be a viable treatment option in SCI. Research has suggested that cell transplantation to the damaged spinal cord is capable of producing new neural connections at the level of injury and improved locomotion [3–5]. There are different populations of stem cells, but they all fall into two pluripotent and multipotent groups. These cells have a continuous self-renewal capacity and can differentiate into somatic cells [6].

Much attention has now been paid to the use of adult stem cells, most commonly mesenchymal cells. There are various sources for these types of cells in the body, but adipose-derived mesenchymal stem cells (ADSCs) are one of the best sources available because they are easy to access and have excellent proliferation and differentiation properties. ADSCs are multipotent and can differentiate into mesenchymal and non-mesenchymal classes [7–9]. The cell population derived from adipose tissue is of mesenchymal origin and has low impurities of endothelial cells, smooth muscle cells, and pericytes. ADSCs enter the aging phase later than other cells, even after passing several passages, and can differentiate into adipogenic, osteogenic, chondrogenic, myogenic, and neurogenic cells [9–11]. These capabilities make them a proper candidate for SCI.

The characteristics and differentiation ability of ADSCs in vivo and in vitro have been widely described in the literature, but yet, there is no consensus on the neuronal healing ability and recovery of neurological symptoms after their transplantation following SCI. Therefore, the present systematic review and meta-analysis aimed to evaluate the effect of transplantation of ADSCs on the improvement of locomotion in preclinical models of SCI.

## Method

### Study design

This study is a systematic review and meta-analysis, conducted based on the Preferred Reporting Items for Systematic Reviews and Meta-Analyses (PRISMA) [12]. This study was not registered.

### PICO definition and eligibility criteria

The description of PICO (Problem, Intervention, Comparison, Outcome) in the current study is as follows: the problem (P) included the rats with SCI. The intervention (I) was the transplantation of ADSCs. The comparison (C) compares the outcome in the treatment group with the result of the SCI group without treatment, and the interested outcome of the study (O) was the improvement of animal locomotion.

Rat studies on SCI which were treated with ADSCs were included. Exclusion criteria included not having control group, not evaluating motor function recovery based on behavioral assessment test, using animal species other than rat, not reporting the number of transplanted cells, insufficient explanation about isolation and preparation of stem cells, review articles, use of combination therapy, use of genetically modified ADSCs, use of induced pluripotent ADSCs, and human trials.

### Search strategy

The keywords related to “adipose-derived stem cell” and “spinal cord injury” were used to perform a comprehensive search on MEDLINE, Embase, Scopus, and Web of Science until the end of July 2019. Search queries in the databases are depicted in the Additional file 1. Also, a manual search was done in the bibliography of related articles and the Google Scholar search engine. Reference tracking and citation tracking of eligible studies were other strategy to achieve more relevant articles.

### Study selection and data collection

After eliminating duplicate records, two independent researchers performed the initial screening studying titles and abstracts. Next, full texts of the potentially relevant articles were assessed and were selected based on the inclusion and exclusion criteria. Any disagreement was then resolved by discussion with a third researcher.

The extracted data included study design information, animals’ age/weight and gender, SCI induction model, number of transplanted ADSCs, time interval between SCI and treatment, severity of injury, location of injury, transplantation type, number of studied animals, and the motor function score. SCI induction models included contusion (dropping a source of weight on the exposed spinal cord of animal model) [13], compression (clipping the exposed spinal cord of animal model) [14], hemisection (cutting the dorsal and ventral columns of the exposed spinal cord of animal model) [15], and crush (hitting the spine using a blower) [16] injury models. If the required data were not recorded in an article, the corresponding author was contacted. Majority of the studies reported the outcome of the intervention in several stages, so the last evaluation time was published in the current meta-analysis. In cases when the results were reported in graphs, Sistrom and Mergo data extraction method was used [17].

### Risk of bias assessment

Quality assessment was evaluated using instructions proposed by Hasannejad et al. [18]. This tool includes 15 items regarding study design, animal characteristics, methodological quality of study, and analysis. The items are animals’ species, using appropriate tests, severity of SCI induction, spinal level of SCI, age/weight of animals, number of animals per group, designation of strain, definition of control, description of statistical analysis, regulation and ethics, bladder expression of animals after SCI, blindness of assessor, genetic background of the included animals, method of allocation to treatments, and attrition.

Two independent reviewers assessed the included studies and determined the risk of bias of each item. Disagreements were resolved by discussion with a third researcher. No recommendations exist for the overall risk of bias score in Hassanejad et al.’s study. Therefore, we defined overall risk of bias consulting with an expert. Accordingly, the presence of at least one fetal error in the methodological approach was defined as high risk and presence of bias in other items without any fetal errors was considered as some concern of bias. Low risk of bias was scored when all items were low risk. Lack of blinding of assessor, not using standard test for assessment of locomotion, and not reporting the severity and level of SCI were considered as fetal errors.

### Data synthesis

Studies were summarized based on locomotion recovery, and data were recorded as mean ± standard deviation (SD). Since most of the animal studies reported standard error of mean (SEM) instead of SD, we calculated SD from SEM (SD = SEM × the square root of sample size in each group). For each separate experiment, the number of animals in each group, mean, and SD were recorded.

### Statistical analysis

Statistical analyses were performed using STATA 14.0 statistical software. Our previous meta-analyses showed that the efficacy of stem cells therapy may vary among different methodological designs, such as diversities in animal species, number of transplanted stem cells, and type of graft [4, 19, 20]. Since the design of the included studies is heterogeneous, from a methodological point of view, a random effect model was used to analyze data. Heterogeneity between the studies was evaluated using the statistic *I*^2^ and chi-squared test. In cases of heterogeneity, subgroup analysis was performed to determine the source of heterogeneity. Finally, the study results were pooled, and the overall effect size was presented. This effect size is calculated as standardized mean difference (SMD) with 95% confidence interval (95% CI). Moreover, a sensitivity analysis was performed using leave-one-out approach to assess any individual study’s effect on the pooled effect size. In addition, we performed a sensitivity analysis according to the overall risk of bias score. Publication bias was identified using Egger’s tests [21].

## Results

### Characteristics

**Fig. 1:**
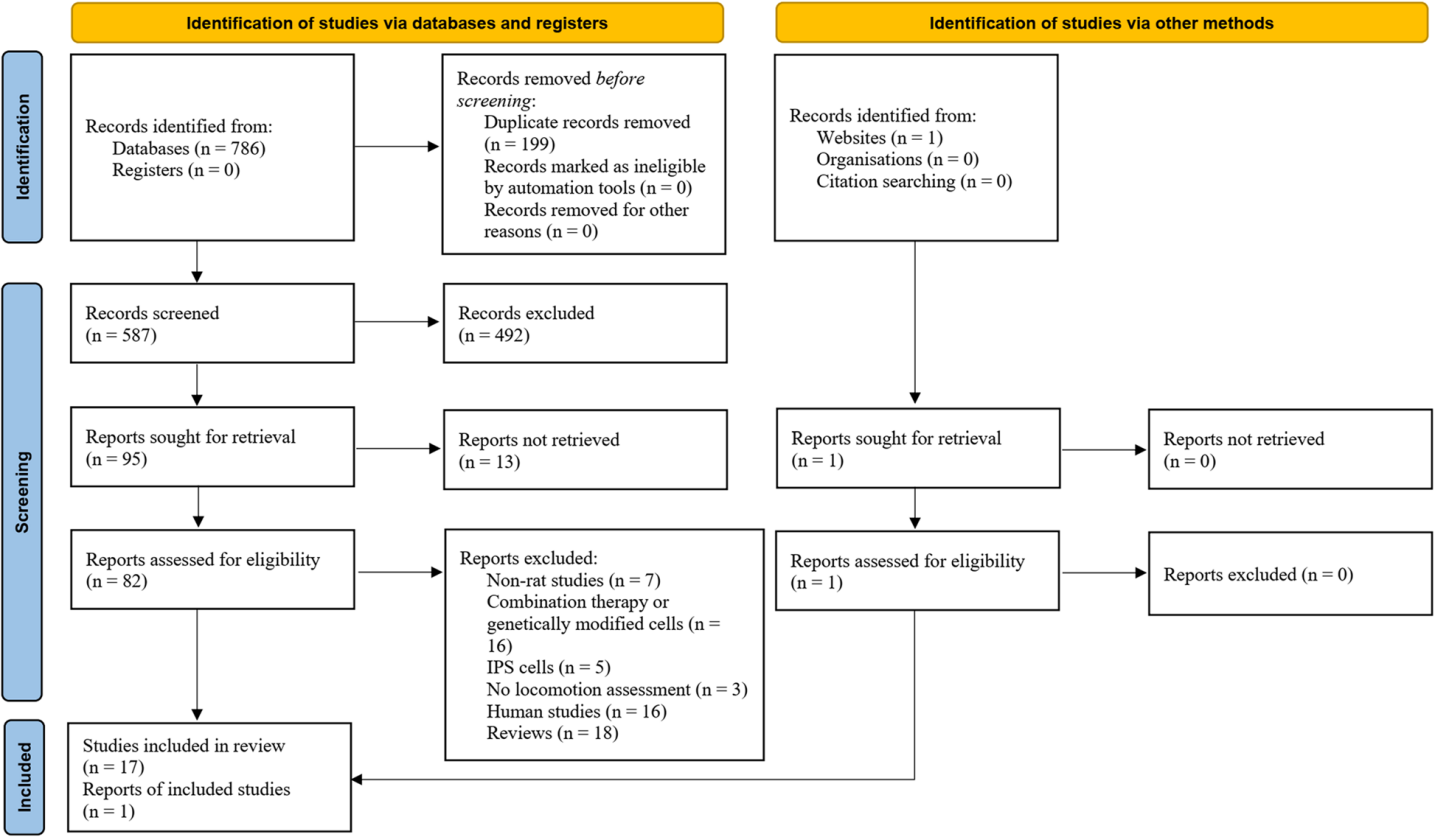
PRISMA flow diagram of present review. Adopted from PRISMA 2020 statement [12]

The search came up with 588 non-duplicate results. After initial screening and studying full texts of the articles, 18 articles were included in the current meta-analysis [13–16, 22–35] (Fig. 1). One of the articles presented two different sets of data [14], so the results from 19 unique experiments were analyzed. Two studies were in Chinese [33, 34], and 16 articles were in English [13–16, 22–32, 35]. Experiments were performed on 567 rats (275 rats in the SCI group and 292 rats in ADSCs-treated groups). Eight studies used the contusion model, and five studies used a compression model to create SCI. Injury severity was moderate in 11 articles and severe in seven studies. The time interval between SCI and treatment administration was between 0 and 14 days. SCI location was the thoracic region in 11 studies. Thirteen studies used intrathecal/intraspinal ADSC administration, and five studies used intravenous administration. The number of transplanted cells varied between 1 × 10^5^ and 2.5 × 10^6^ cells. Table 1 summarizes the characteristics of the included studies.

**Table 1:** Summary of eligible studies

**Author; year**	**Gender; strain; species**	**Sample size** **SCI; treated**	**Model**	**Severity of injury**	**Injury to treatment interval (day)**	**Location of injury**	**Immunosuppressive; antibiotic**	**Transplantation type**	**Number of transplanted cell**	**Follow-up (week)**
Abdanipour; 2014 [13]	Female; Sprague–Dawley; rat	8; 8	Contusion	Moderate	7	Lumbar	No; Yes	IS/IT	3 × 10^5^	12
Aras; 2016 [14]	Female; Wistar; rat	7; 14	Compression	Severe	0, 9	Thoracolumbar	No; No	IS/IT	3 × 10^5^	4
da Silva; 2014 [22]	NR; Wistar; rat	12; 12	Compression	Moderate	7, 14	Thoracic	No; Yes	IV	1.2 × 10^6^	13
Kang; 2006 [15]	Female; Wistar; rat	3; 3	Hemisection	Severe	3	Thoracolumbar	No; No	IV	2 × 10^6^	4
Menezes; 2014 [23]	Female; Sprague–Dawley; rat	9; 9	Compression	Moderate	0	Thoracic	No; Yes	IS/IT	NR	8
Min; 2017 [24]	Female; Sprague–Dawley; rat	5; 8	Contusion	Severe	3, 7, 14	Thoracic	No; Yes	IS/IT	1 × 10^6^	8
Oh; 2012 [25]	Male; Sprague–Dawley; rat	10; 10	Compression	Severe	0	Thoracic	No; No	IS/IT	1 × 10^5^	6
Ohta; 2017 [26]	Female; Sprague–Dawley; rat	15; 20	Contusion	Moderate	8	Thoracolumbar	No; Yes	IV	2.5 × 10^6^	7
Ohta; 2018 [27]	Female; Sprague–Dawley; rat	8; 10	Contusion	Moderate	8	Thoracolumbar	No; Yes	IV	2.5 × 10^6^	6
Rosado; 2017 [28]	Male; Wistar; Rat	50; 50	Compression	Moderate	0	Thoracic	No; Yes	IV	1 × 10^6^	3
Sarveazad; 2014 [30]	Male; Wistar; rat	6; 6	Contusion	Moderate	7	Thoracic	No; Yes	IS/IT	1 × 10^6^	8
Sarveazad; 2017 [29]	Male; Wistar; rat	6; 6	Contusion	Moderate	7	Thoracic	No; Yes	IS/IT	1 × 10^6^	9
Tang; 2016 [31]	Female; Sprague–Dawley; rat	16; 16	Contusion	Severe	9	Thoracic	No; No	IS/IT	1 × 10^6^	8
Wang; 2017 [16]	Male; Sprague–Dawley; rat	76; 76	Crush	Moderate	0	Thoracic	No; Yes	IS/IT	1 × 10^6^	4
Zhang; 2009 [32]	Male; Sprague–Dawley; rat	12; 12	Contusion	Moderate	7	Thoracic	No; No	IS/IT	NR	11
Zhang; 2014 [33]	Male; Sprague–Dawley; rat	15; 15	Crush	Moderate	0	Thoracolumbar	Yes; Yes	IS/IT	2 × 10^5^	3
Zheng; 2017 [34]	Female; Sprague–Dawley; rat	12; 12	Hemisection	Severe	0	Thoracolumbar	No; No	IS/IT	1 × 10^6^	7
Zhou; 2013 [35]	Female; Sprague–Dawley; rat	5; 5	Hemisection	Severe	0	Thoracic	Yes; No	IS/IT	2 × 10^5^	4

*IS* Intraspinal, *IT* Intrathecal, *IV* Intravenous, *NR* Not reported

### Risk of bias assessment and publication bias

**Fig. 2:**
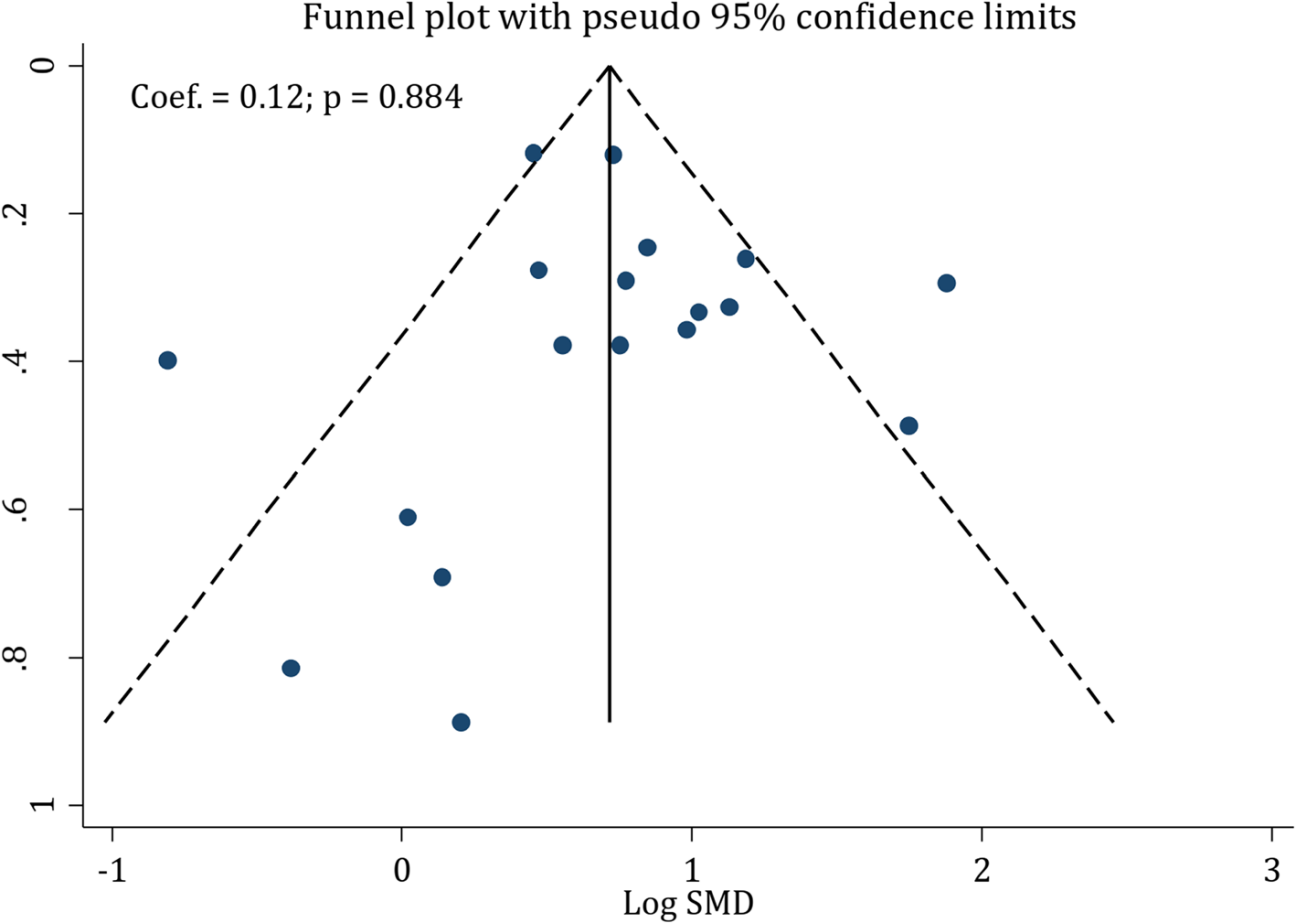
Publication bias among included studies. There is no evidence of publication bias (*p* = 0.884)

Quality control of the studies demonstrated that the risk of bias in attrition bias and blinding of the assessor sections was high in 14 articles and eight articles, respectively. Bladder expression was not reported in 6 articles (high risk). In other parts, the majority of the studies had a low risk of bias (Table 2). Overall risk of bias was high risk in 8 studies, some concern in 9 studies and low risk in 1 study. Finally, analyses showed that there was no publication bias in the current meta-analysis (*p* = 0.884) (Fig. 2).

**Table 2:**
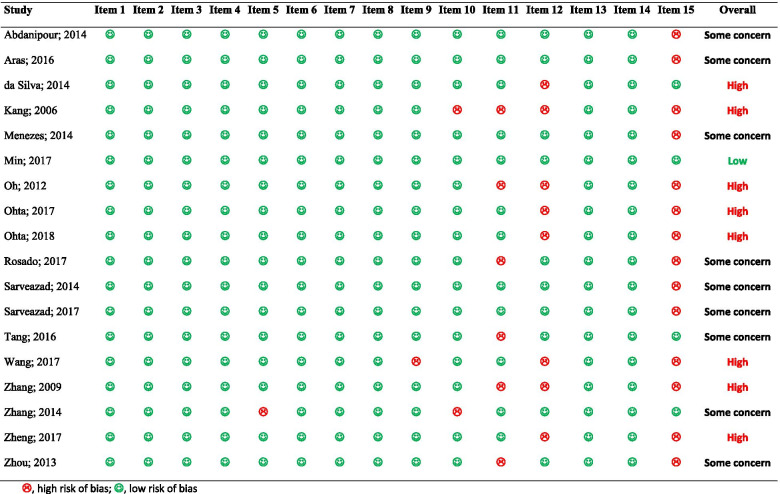
Risk of bias assessment of included studies

Item 1, species; Item 2, using appropriate tests; Item 3, severity of injury; Item 4, level of injury; Item 5, age/weight; Item 6, number of animals per group; Item 7, designation of strain; Item 8, definition of control; Item 9, description of statistical analysis; Item 10, regulation and ethics; Item 11, bladder expression; Item 12, blindness of assessor; Item 13, genetic background; Item 14, method of allocation to treatments; Item 15, attrition

### The effect of ADSCs on locomotion after SCI

**Fig. 3:**
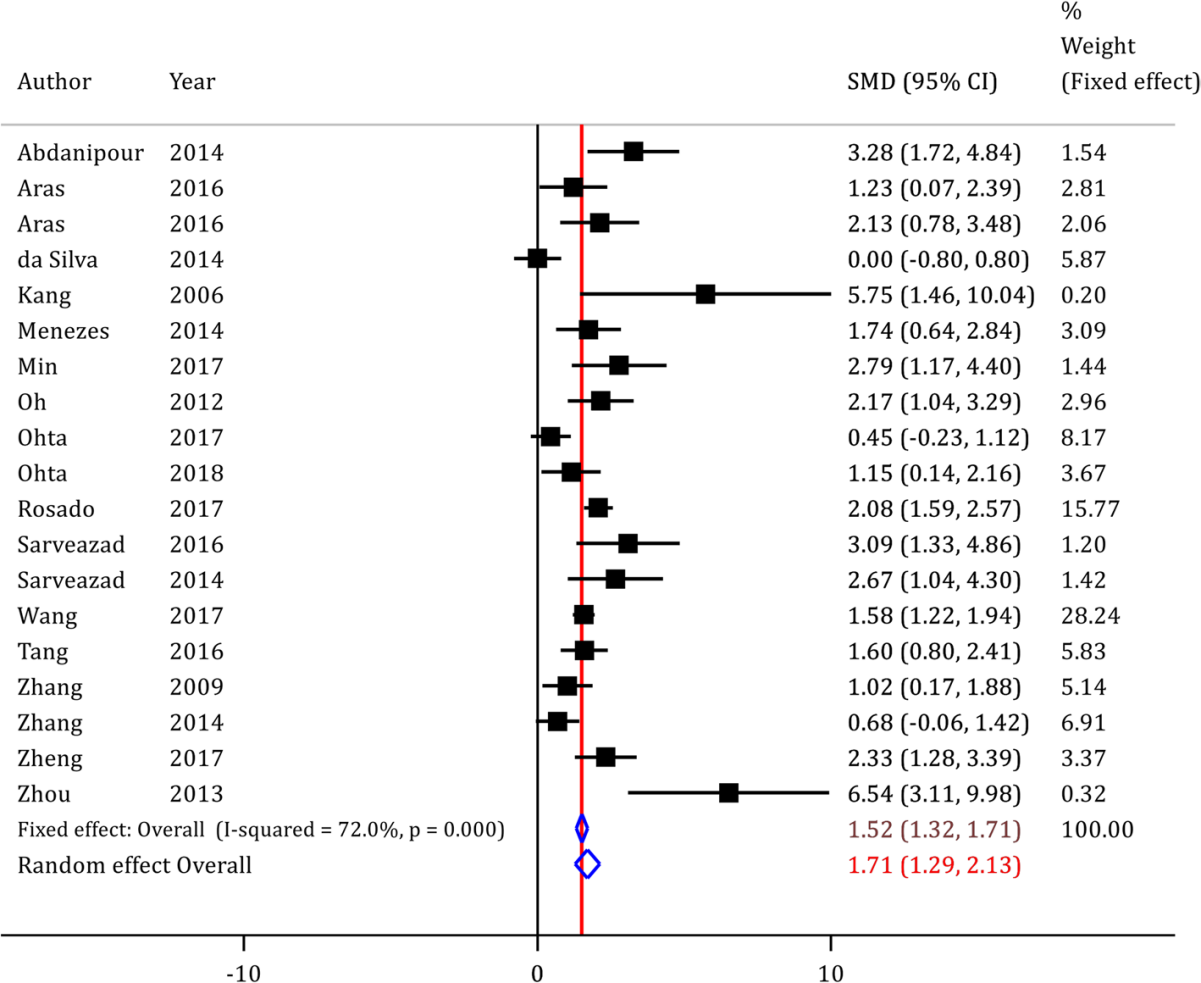
Forest plot of efficacy of adipose tissue derived stem cell on locomotion of spinal cord injured animals. CI, confidence interval; SMD, standardized mean difference

Random effect analysis demonstrated that ADSC transplantation can improve locomotion in rats following SCI (SMD = 1.71; 95% CI 1.29 to 2.13; *p* < 0.0001). We performed an additional analysis based on fixed effect model. According to the fixed effect model, the analysis showed that the pooled SMD of ADSCs on locomotion recovery after SCI is 1.52 (95% CI 1.32 to 1.71). Therefore, both models show a significant beneficial effect of ADSCs administration on locomotion after SCI (Fig. 3).

Importantly, considerable heterogeneity was observed between the studies (*I*^2^ = 72.0%; p < 0.0001). Hence, a subgroup analysis was performed (Fig. 3).

Subgroup analysis depicted that the severity of the injury and the number of transplanted cells were the two main sources of heterogeneity between the studies (Table 3). Univariate meta-regression showed that differences in injury model, the severity of SCI, treatment phase, injury location, number of transplanted cells, route of administration, type of graft, and follow-up duration did not have a significant effect on the efficacy of ADSCs improving locomotion following SCI (*p*_for odds ratios_ > 0.05). In other words, ADSCs seem to improve motor function in every setting (Table 3).

**Table 3:** Subgroup analysis of adipose tissue derived stem cells on motor function recovery after spinal cord injury

**Subgroup**	**Number of experiments**	**Heterogeneity (*p* value)**	**SMD (95% CI)**	***P* value**	**OR (95% CI)**	***P* value**
**Injury model**						
Contusion/compression	14	71.1% (< 0.0001)	1.65 (1.15*–*2.15)	< 0.0001	*Ref*	*–*
Crush	2	71.9% (< 0.0001)	1.19 (0.32*–*2.06)	0.008	0.61 (0.14–2.64)	0.481
Hemisection	3	72.0% (< 0.0001)	4.45 (1.33*–*7.57)	0.005	6.26 (0.94–41.65)	0.306
**Severity of SCI**						
Moderate	11	77.3% (*p* < 0.0001)	1.42 (0.91–1.94)	< 0.0001	*Ref*	*–*
Sever	8	48.8% (*p* = 0.057)	2.23 (1.55–2.91)	< 0.0001	2.30 (0.76–6.93)	0.129
**Treatment phase**						
Immediate and acute	10	64.7% (*p* = 0.003)	1.89 (1.38–2.40)	< 0.0001	*Ref*	*–*
Sub-acute	9	73.8% (*p* < 0.0001)	1.50 (0.82–2.18)	< 0.0001	0.61 (0.20–1.85)	0.362
**Injury location**						
Thoracic	11	72.3% (< 0.0001)	1.81 (1.28–2.33)	< 0.0001	*Ref*	*–*
Thoracolumbar and lumbar	8	70.9% (0.001)	1.29 (0.85–2.32)	< 0.0001	0.76 (0.24–2.42)	0.620
**Number of transplanted cell**						
≤ 5.0 × 10^5^	6	75.4% (0.001)	2.14 (1.07–3.20)	< 0.0001	*Ref*	*–*
5.1 × 10^5^ to 1.0 × 10^6^	6	28.1% (0.224)	1.91 (1.55–2.26)	< 0.0001	1.10 (0.25–4.72)	0.890
> 1.0 × 10^6^	5	75.0% (0.003)	1.17 (0.14–2.19)	0.026	0.40 (0.09–1.97)	0.237
**Route of administration**						
Intraspinal/intrathecal	14	57.0% (0.004)	1.89 (1.46–2.32)	< 0.0001	*Ref*	*–*
Intravenous	5	86.7% (< 0.0001)	1.18 (0.11–2.25)	0.031	0.43 (0.13–1.35)	0.138
**Type of graft**						
Allograft	11	62.5% (0.003)	1.64 (1.22–2.05)	< 0.0001	*Ref*	*–*
Xenograft	7	80.7% (< 0.0001)	1.91 (0.86–2.95)	< 0.0001	1.40 (0.44–4.41)	0.542
Autograft	1	NA	NA	NA	NA	NA
**Follow-up duration**						
3–4 weeks	7	72.7% (0.001)	1.78 (1.12–2.44)	< 0.0001	*Ref*	*–*
5–7 weeks	6	75.0% (0.007)	1.46 (0.51–2.41)	0.003	0.65 (0.13–3.19)	0.573
8–13 weeks	7	74.4% (< 0.0001)	1.85 (1.04–2.65)	< 0.0001	0.93 (0.23–3.69)	0.911
**Risk of bias**						
Low risk	1	NA	NA	NA	NA	NA
Some concern	10	64.6% (0.003)	2.02 (1.43–2.61)	< 0.001	*Ref*	*–*
High risk	8	75.9 (< 0.001)	1.28 (0.66–1.90)	< 0.001	2.10 (0.71–6.21)	0.168

Severity of injury was categorized based on the definition given in the article by Cheriyan et al. [36]

*CI* Confidence interval, *NA* Not applicable due to limited number of studies in the category, *OR* Odds ratio, *Ref*. Reference category, *SCI* Spinal cord injury, *SMD* Standardized mean difference

### Sensitivity analysis

**Fig. 4:**
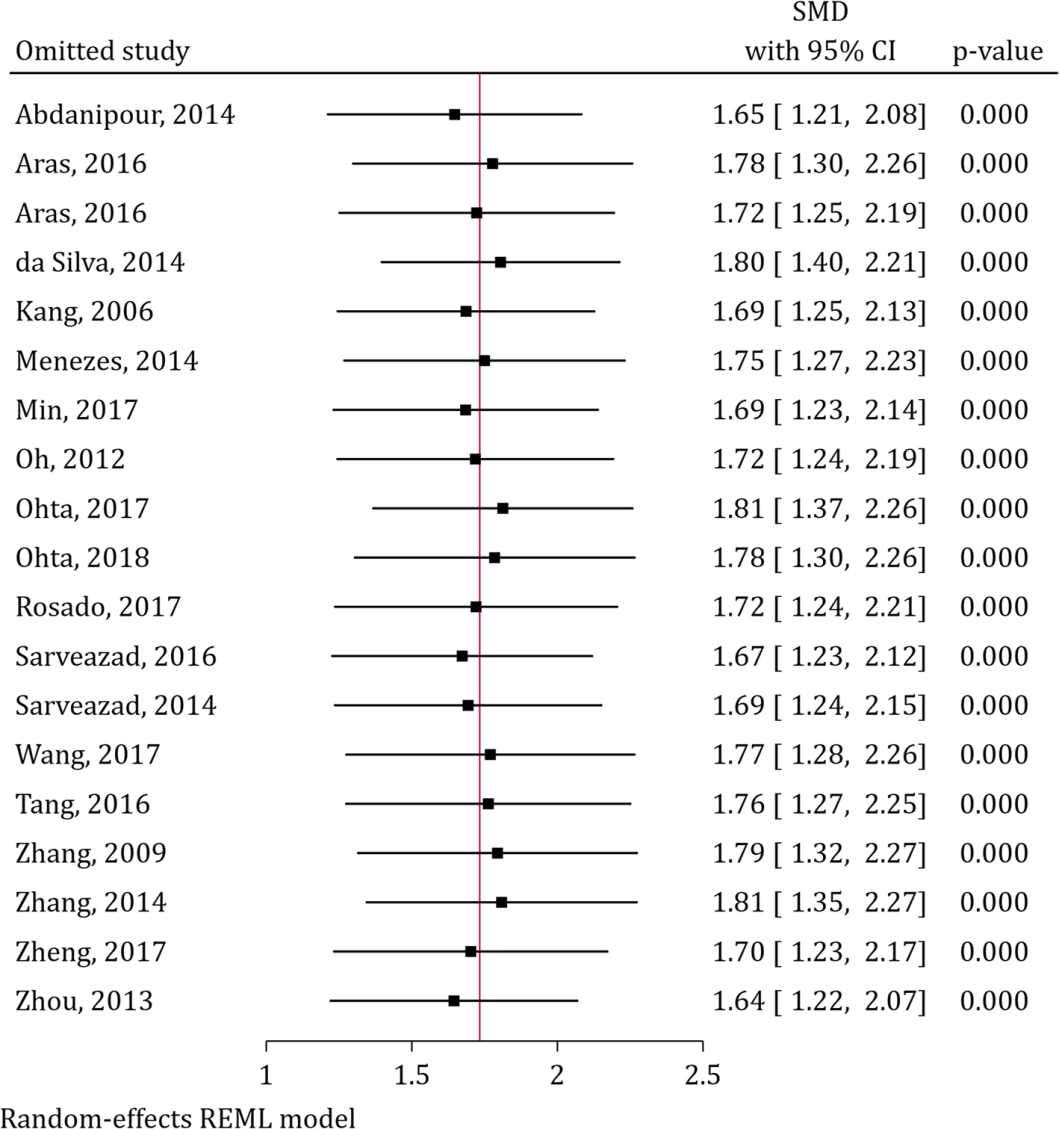
Leave-one-out sensitivity analysis for assessment of individual study effect on effect size

We used leave-one-out sensitivity analysis to explore any individual studies’ effect on the pooled SMD. The analysis showed that excluding any of the included articles does not statistically affect the pooled SMD (Fig. 4). In addition, another sensitivity analysis based on the risk of bias score depicted that the pooled SMD of ADSCs transplantation after SCI in high risk of bias studies (SMD = 1.28; 95% CI 0.66–1.90) did not significantly differ from studies having some concern risk of bias (SMD = 2.02; 95% CI 1.43–2.61; odds ratio = 2.10; 95% CI 0.71–6.21; *p* = 0.168; Table 3).

## Discussion

The present study aimed to summarize the evidence regards the efficacy of ADSC transplantation on locomotor recovery following SCI in rats. Overall, the current meta-analysis showed that the ADSCs could significantly improve locomotor function following SCI in rats. This improvement is not affected by injury model, the severity of SCI, treatment phase, injury location, number of transplanted cells, route of administration, type of graft, and follow-up duration.

Regarding the injury model, although present meta-analysis did not show a significant relationship between the injury model and ADSCs efficacy, it should be noted that the number of studies included for the crush (2 studies) and hemisection (3 studies) models is low. Therefore, a definite conclusion on the relationship of motor function improvement after transplantation of ADSCs and injury model needs further experimental investigations.

ADSCs have anti-apoptotic, anti-inflammatory, anti-fibrotic, immunomodulatory, and angiogenesis properties due to the secretion of various cytokines [37]. These features of ADSCs, along with accessibility and abundance, make ADSCs, a suitable candidate for neural tissue repair [38]. ADSCs induce axonal regeneration, synaptogenesis, extracellular matrix (ECM) rearrangement, and angiogenesis through the expression of cytokines such as stem cell factor, neural growth factor, brain-derived neurotrophic factor, matrix metalloproteinase, and vascular endothelial growth factor and ultimately improve locomotion recovery [26, 39–42]. Antonic et al.’s systematic review and meta-analysis on stem cell transplantation in traumatic SCI in animal studies also show that stem cells after SCI improve motor function [43]. They showed that in 70% of the studies, the Basso, Beattie, and Bresnahan (BBB) locomotor scale used to assess motor status. They concluded that studies using other criteria besides the BBB reported less efficacy for stem cell therapy. This issue could be considered as a potential source of bias. Also, Antonic et al. showed that the blinding status of the observer was a significant issue when a behavioral test, such as the BBB, was performed. They reported that studies examining locomotor outcomes would fail to reach a definitive conclusion about efficacy if they did not report blinding. Locomotion recovery findings and blinding bias in our study are consistent with the results of Antonic et al.

In the randomized clinical trials, blinding is far more important than animal studies. Systematic review and meta-analysis by Xu et al. in 2019 showed that mesenchymal stem cell transplantation in SCI patients did not affect improving motor function [44]. A systematic review and meta-analysis of Fan et al. in 2017 investigated the safety and efficacy of stem cell transplantation in SCI patients. All studies included in this meta-analysis (except one study) used mesenchymal stem cells. The results of this study showed that stem cell transplantation is safe in SCI patients but has no efficacy in improving motor function. In their meta-analysis, only two of 10 articles had no blinding [45]. Thus, at least part of the reason for the discrepancy in the effect of stem cells on improving motor function between meta-analysis of animal studies and clinical trials could be related to the application of various tests in the evaluation of motor improvement and blinding compliance in most clinical trials. However, the leading cause is the complexity of the nervous system and the involvement of far more complex circuits in the human motor system.

Worth to mention that sometimes, choosing a wrong statistical model may lead to inaccurate results, and using fixed or random effect models under different meta-analytical scenarios may result in different findings. Although we decided to use random effect model to pool the results of included studies, using fixed effect or random effect model did not change the overall results. The pooled SMD of fixed effect model and random effect model for effect of ADSCs on locomotion after SCI are 1.52 (95% CI 1.32 to 1.71) and 1.71 (95% CI 1.29 to 2.13), respectively. Therefore, even though we used a random effect model based on our expectation of heterogeneity among included studies, both models showed a significant beneficial effect of ADSCs on locomotion after SCI.

## Conclusion

We conclude that any number of ADSCs by any prescription routes can improve locomotion recovery if administered in an SCI animal model, at any phase of SCI, with any severity. But given the remarkable bias about blinding, it is tough to clinical translation of the present results, because in addition to the complexity of the nervous system and the involvement of far more complex motor circuits in the human, blinding compliance and motor outcome assessment tests in animal studies and clinical trials are significantly different.

## Supplementary Information

**Additional file 1.** Search query for databases.

## Data Availability

All data generated or analyzed during this study are included in this published article.

## Abbreviations

ADSC: Adipose tissue-derived stem cells
BBB: Basso, Beattie, and Bresnahan
CI: Confidence interval
ECM: Extracellular matrix
IS: Intraspinal
IT: Intrathecal
IV: Intravenous
NA: Not applicable
NR: Not reported
OR: Odds ratio
PICO: Problem, Intervention, Comparison, Outcome
PRISMA: Preferred Reporting Items for Systematic Reviews and Meta-Analyses
Ref.: Reference category
SCI: Spinal cord injury
SMD: Standardized mean difference
