# Supplementary material for: Efficacy of adipose tissue-derived stem cells in locomotion recovery after spinal cord injury: a systematic review and meta-analysis on animal studies
Source: Syst Rev. 2021 Jul 31;10:213. doi: 10.1186/s13643-021-01771-w (PMC8325264; doi:10.1186/s13643-021-01771-w)
Supplement: Supplementary file 1 — Additional file 1. Search query for databases. [file 13643_2021_1771_MOESM1_ESM.docx]

**Additional file 1: Search query for databases**

1. "Mesenchymal Stem Cells"[mh] OR Adipose-derived Stem Cells[TIAB] OR Human Adipose-derived Stem Cells[TIAB] OR (potential[All Fields] AND adipose stem cells[TIAB]) OR (("adult stem cells"[MeSH Terms] OR ("adult"[All Fields] AND "stem"[All Fields] AND "cells"[All Fields]) OR "adult stem cells"[All Fields]) AND derived[All Fields] AND adipose tissue[TIAB]) OR (("stem cells"[MeSH Terms] OR ("stem"[All Fields] AND "cells"[All Fields]) OR "stem cells"[All Fields]) AND fat[TIAB]) OR (("stem cells"[MeSH Terms] OR ("stem"[All Fields] AND "cells"[All Fields]) OR "stem cells"[All Fields]) AND adipose tissue[TIAB]) OR Adipose Stem Cell[TIAB] OR (Fat[All Fields] AND tissue stem cells[TIAB]) OR (("stem cells"[MeSH Terms] OR ("stem"[All Fields] AND "cells"[All Fields]) OR "stem cells"[All Fields]) AND adipose tissue[TIAB]) OR (("adipose tissue"[MeSH Terms] OR ("adipose"[All Fields] AND "tissue"[All Fields]) OR "adipose tissue"[All Fields]) AND Derived[All Fields] AND Multipotent Mesenchymal Stromal Cells[TIAB]) OR (Brown[All Fields] AND Adipose Tissue Derived Stem Cells[TIAB]) OR Autologous Adipose Tissue Derived Mesenchymal Stem Cells[TIAB] OR Adipose tissue stem cells[TIAB] OR Adipose Tissue-Derived Stem Cells[TIAB] OR (Stromal[All Fields] AND ("stem cells"[MeSH Terms] OR ("stem"[All Fields] AND "cells"[All Fields]) OR "stem cells"[All Fields]) AND Human Adipose Tissue[TIAB]) OR Adipose-Derived Mesenchymal Stem Cells[TIAB] OR (("humans"[MeSH Terms] OR "humans"[All Fields] OR "human"[All Fields]) AND ("mesenchymal stem cells"[MeSH Terms] OR ("mesenchymal"[All Fields] AND "stem"[All Fields] AND "cells"[All Fields]) OR "mesenchymal stem cells"[All Fields]) AND derived[All Fields] AND adipose tissue[TIAB]) OR Adipose tissue stem cells[TIAB] OR (("cells"[MeSH Terms] OR "cells"[All Fields]) AND fat[TIAB]) OR Adipose derived Mesenchymal stem cells[TIAB] OR (("mesenchymal stem cells"[MeSH Terms] OR ("mesenchymal"[All Fields] AND "stem"[All Fields] AND "cells"[All Fields]) OR "mesenchymal stem cells"[All Fields]) AND Adipose Tissue[TIAB]) OR Adipose tissue stem cells[TIAB] OR Adipose Tissue-Derived Mesenchymal Stem Cells[TIAB] OR Adipose Tissue Stem Cells[TIAB] OR (("stromal cells"[MeSH Terms] OR ("stromal"[All Fields] AND "cells"[All Fields]) OR "stromal cells"[All Fields]) AND adipose tissue[TIAB]) OR ((("stem cells"[MeSH Terms] OR ("stem"[All Fields] AND "cells"[All Fields]) OR "stem cells"[All Fields]) AND derived[All Fields] AND mesenchymal[All Fields] AND ("tissues"[MeSH Terms] OR "tissues"[All Fields])) AND TIAB[All Fields]) OR Adipose-derived adult stem cells[TIAB] OR (("stem cells"[MeSH Terms] OR ("stem"[All Fields] AND "cells"[All Fields]) OR "stem cells"[All Fields]) AND mouse adipose tissue[TIAB]) OR Adipose tissue mesenchymal stem cells[TIAB] OR Adipogenic[TIAB] OR Adipogenesis[TIAB] OR Adiposytes[TIAB]
2. "spinal cord injuries"[mh] OR spinal cord injury[tiab] OR spinal cord contusion[tiab] OR spinal cord hemisection[tiab] OR spinal cord transection[tiab] OR cervical spine injury[tiab] OR Spinal compression[tiab] OR spinal cord trauma[tiab] OR trauma, spinal cord[tiab] OR injured spinal cord[tiab] OR spinal cord injured[tiab] OR spinal cord injuries[tiab] OR nerve transection[tiab]
3. #1 AND #2

**Embase**

1. 'adipose derived stem cell'/exp OR 'adipose derived stem cell' OR 'adipose-derived stem cells':ab,ti OR 'human adipose-derived stem cells':ab,ti OR 'potential of adipose stem cells':ab,ti OR 'adult stem cells derived from adipose tissue':ab,ti OR 'stem cells from fat':ab,ti OR 'adipose stem cell':ab,ti OR 'fat tissue stem cells':ab,ti OR 'stem cells from adipose tissue':ab,ti OR 'adipose tissue derived multipotent mesenchymal stromal cells':ab,ti OR 'brown adipose tissue derived stem cells':ab,ti OR 'autologous adipose tissue derived mesenchymal stem cells':ab,ti OR 'adipose tissue-derived stem cells':ab,ti OR 'stromal stem cells from human adipose tissue':ab,ti OR 'adipose-derived mesenchymal stem cells':ab,ti OR 'human mesenchymal stem cells derived from adipose tissue':ab,ti OR 'cells from fat':ab,ti OR 'adipose derived mesenchymal stem cells':ab,ti OR 'mesenchymal stem cells from adipose tissue':ab,ti OR 'adipose tissue‐derived mesenchymal stem cells':ab,ti OR 'adipose tissue stem cells':ab,ti OR 'stromal cells from the adipose tissue':ab,ti OR 'stem cells derived from various mesenchymal tissues:':ab,ti OR 'adipose-derived adult stem cells':ab,ti OR 'stem cells from mouse adipose tissue':ab,ti OR 'adipose tissue mesenchymal stem cells':ab,ti
2. 'spinal cord injury'/exp OR 'spinal cord contusion'/exp OR 'spinal cord hemisection'/exp OR 'spinal cord transsection'/exp OR 'cervical spine injury'/exp OR 'spinal compression':ab,ti OR 'spinal cord trauma':ab,ti OR 'trauma, spinal cord':ab,ti OR 'injured spinal cord':ab,ti OR 'spinal cord injured':ab,ti OR 'spinal cord injuries':ab,ti OR 'nerve transection':ab,ti
3. #1 AND #2

**Scopus:**

1. ( TITLE-ABS-KEY ( "spinal cord injury" )  OR  TITLE-ABS-KEY ( "spinal cord contusion" )  OR  TITLE-ABS-KEY ( "spinal cord hemisection" )  OR  TITLE-ABS-KEY ( "spinal cord transection" )  OR  TITLE-ABS-KEY ( "cervical spine injury" )  OR  TITLE-ABS-KEY ( "spinal cord injury" )  OR  TITLE-ABS-KEY ( "spinal cord contusion" )  OR  TITLE-ABS-KEY ( "spinal cord hemisection" )  OR  TITLE-ABS-KEY ( "spinal cord transection" )  OR  TITLE-ABS-KEY ( "cervical spine injury" )  OR  TITLE-ABS-KEY ( "Spinal compression" )  OR  TITLE-ABS-KEY ( "spinal cord trauma" )  OR  TITLE-ABS-KEY ( "trauma, spinal cord" )  OR  TITLE-ABS-KEY ( "injured spinal cord" )  OR  TITLE-ABS-KEY ( "spinal cord injured" )  OR  TITLE-ABS-KEY ( "spinal cord injuries" )  OR  TITLE-ABS-KEY ( "nerve transection" ) )
2. ( TITLE-ABS-KEY ( "Adipose-derived Stem Cells" )  OR  TITLE-ABS-KEY ( "Human Adipose-derived Stem Cells" )  OR  TITLE-ABS-KEY ( "Adult Stem Cells derived from adipose tissue" )  OR  TITLE-ABS-KEY ( "Stem cells from adipose tissue" )  OR  TITLE-ABS-KEY ( "Adipose Stem Cell" )  OR  TITLE-ABS-KEY ( "Fat tissue stem cells " )  OR  TITLE-ABS-KEY ( "Stem cells from adipose tissue " )  OR  TITLE-ABS-KEY ( "Adipose Tissue Derived Multipotent Mesenchymal Stromal Cells" )  OR  TITLE-ABS-KEY ( "Autologous Adipose Tissue Derived Mesenchymal Stem Cells" )  OR  TITLE-ABS-KEY ( "Adipose tissue stem cells" )  OR  TITLE-ABS-KEY ( "Adipose Tissue-Derived Stem Cells" )  OR  TITLE-ABS-KEY ( "Stromal Stem Cells from Human Adipose Tissue" )  OR  TITLE-ABS-KEY ( "Adipose-Derived Mesenchymal Stem Cells" )  OR  TITLE-ABS-KEY ( "Human mesenchymal stem cells derived from adipose tissue" )  OR  TITLE-ABS-KEY ( "Adipose tissue stem cells" )  OR  TITLE-ABS-KEY ( "Adipose derived Mesenchymal stem cells" )  OR  TITLE-ABS-KEY ( "Mesenchymal Stem Cells from Adipose Tissue" ) )
3. #1 AND #2

**Web of Science**

1. TS=("Adipose-derived Stem Cells" OR "Adipose-derived Stem Cells" OR "Human Adipose-derived Stem Cells" OR "The potential of adipose stem cells" OR "Adult Stem Cells derived from adipose tissue" OR "Stem cells from fat" OR "Stem cells from adipose tissue" OR "Adipose Stem Cell" OR "Fat tissue stem cells " OR "Stem cells from adipose tissue " OR "Adipose Tissue Derived Multipotent Mesenchymal Stromal Cells" OR "Brown Adipose Tissue Derived Stem Cells" OR "Autologous Adipose Tissue Derived Mesenchymal Stem Cells" OR "Adipose tissue stem cells" OR "Adipose Tissue-Derived Stem Cells" OR "Stromal Stem Cells from Human Adipose Tissue" OR "Adipose-Derived Mesenchymal Stem Cells" OR "Human mesenchymal stem cells derived from adipose tissue" OR "Adipose tissue stem cells" OR "Cells from fat" OR "Adipose derived Mesenchymal stem cells" OR "Mesenchymal Stem Cells from Adipose Tissue" OR "Adipose tissue stem cells" OR "Adipose Tissue‐Derived Mesenchymal Stem Cells" OR "Adipose Tissue Stem Cells" OR "Stromal cells from the adipose tissue" OR "Stem cells derived from various mesenchymal tissues" OR "Adipose-derived adult stem cells" OR "Stem cells from mouse adipose tissue" OR "Adipose tissue mesenchymal stem cells")
2. TS=("spinal cord injury" OR "spinal cord contusion" OR "spinal cord hemisection" OR "spinal cord transsection" OR "cervical spine injury" OR "spinal cord injury" OR "spinal cord contusion" OR "spinal cord hemisection" OR "spinal cord transsection" OR "cervical spine injury" OR "Spinal compression" OR "spinal cord trauma" OR "trauma, spinal cord" OR "injured spinal cord" OR "spinal cord injured" OR "spinal cord injuries" OR "nerve transection")
3. #1 AND #2
